# Using graphlet degree vectors to predict atomic displacement parameters in protein structures

**DOI:** 10.1107/S2059798323009142

**Published:** 2023-11-21

**Authors:** Jure Pražnikar

**Affiliations:** a Faculty of Mathematics, Natural Sciences and Information Technologies, University of Primorska, Glagoljaška 8, Koper, Slovenia; b Department of Biochemistry, Molecular and Structural Biology, Institute Jožef Stefan, Jamova 39, Ljubljana, Slovenia; University of Konstanz, Germany

**Keywords:** atomic displacement parameters, graphlet degree vectors, interatomic contacts, macromolecules

## Abstract

In structural biology, atomic displacement parameters, commonly used in the form of *B* values, describe uncertainties in atomic positions. Their distribution over the structure can provide hints on local structural reliability and mobility. A spatial macromolecular model can be represented by a graph whose nodes are atoms and whose edges correspond to all interatomic contacts within a certain distance. Small connected subgraphs, called graphlets, provide information about the wiring of a particular atom. The multiple linear regression approach based on this information aims to predict a distribution of values of isotropic atomic displacement parameters (*B* values) within a protein structure, given the atomic coordinates and molecular packing. By modeling the dynamic component of atomic uncertainties, this method allows the *B* values obtained from experimental crystallographic or cryo-electron microscopy studies to be reproduced relatively well.

## Abstract 2

The components of the graphlet degree vector, which describes the complexity of the wiring of a given atom, can be used in a multiple linear regression model to predict atomic displacement parameters in protein structures.

## 1. Introduction

In experimental methods such as macromolecular crystallo­graphy and cryo-electron microscopy (cryo-EM), uncertainty in atomic positions is described by the atomic displacement parameter (ADP), commonly in the form of a *B* value (Trueblood *et al.*, 1996 ▸; Parthasarathy & Murthy, 1997 ▸; Radivojac *et al.*, 2004 ▸; Carugo, 2018*a* ▸; Sun *et al.*, 2019 ▸). In fact, this parameter includes both the actual atomic mobility, which is the subject of our interest, and variation of the atomic position over the sample, *i.e.* static uncertainties. The ADPs are refined before deposition in the Protein Data Bank (PDB; Berman *et al.*, 2000 ▸). At medium resolution, the ratio of the number of observations to the number of parameters is low and the experimental data are insufficient, so restraints and constraints are needed to refine the coordinates and ADPs. A simple restraint used in modern crystallographic software is that bonded atoms tend to have similar ADPs (Hirshfeld, 1976 ▸; Konnert & Hendrickson, 1980 ▸; Tronrud, 1996 ▸; Merritt, 2011 ▸, 2012 ▸). However, refinement also depends on the crystallo­grapher, who determines the strength of the restraints and the constraints; for example, the minimum and maximum *B* values allowed. The accuracy of ADPs and their maximal values in protein models were the subjects of studies by Carugo (2018*b* ▸, 2022 ▸). These studies showed that very large ADPs were deposited more frequently in the PDB after 2008 and that there has been no improvement in ADP errors over the last two decades.

Recently, Masmaliyeva & Murshudov (2019 ▸) and Masmaliyeva *et al.* (2020 ▸) suggested a very interesting method for analyzing and validating isotropic ADPs. It was shown that the distribution of isotropic ADPs in a protein structure follows a shifted inverse gamma distribution (SIGD), which was defined as

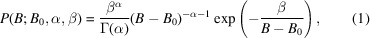

where α, β and *B*_0_ are shape, scale and shift parameters, respectively. Note that *B*_0_ is defined as 90% of the minimum *B* value in the protein model. The statistics for the shape and scale parameters of the SIGD were obtained from a large PDB data set. By estimating the shape and scale parameters of the SIGD, information can be obtained on whether the ADP distribution of the query protein is an outlier that requires further validation. At the same time, this method cannot be used to predict ADPs.

Instead, several methods to predict the isotropic ADPs of macromolecules have been suggested using the amino-acid sequence (Yuan *et al.*, 2005 ▸; Schlessinger & Rost, 2005 ▸; Schlessinger *et al.*, 2006 ▸; Pan & Shen, 2009 ▸), packing density (Halle, 2002 ▸), graph-theory parameters (Jacobs *et al.*, 2001 ▸; Gohlke *et al.*, 2004 ▸; Yin *et al.*, 2011 ▸), elastic networks of C^α^ atoms (Kundu *et al.*, 2002 ▸), local structure-assembly variations (Yang *et al.*, 2016 ▸) and advanced machine-learning algorithms (Bramer & Wei, 2018 ▸).

To our knowledge, none of these methods is widely used in practice. A review of the cited scientific papers shows that (i) some scripts or software are not available, (ii) the prediction is based on the sequence and not on the atomic model and (iii) the predicted *B* values are constant for all atoms in a given residue. To address all of these issues, and to further develop the prediction of *B* values, the method introduced by Weiss (2007 ▸) was chosen, where a linear model was introduced in which the *B* values depend on the parameters of the close atomic contacts. Therefore, by adding parameters describing the local wiring patterns, the linear model was extended into a multiple linear model that is relatively simple and intuitive to construct.

From the perspective of graph theory each contact can be considered as an edge between two nodes (atoms), and the number of contacts per atom is called the node degree in graph theory. This local graph parameter can be used to classify or to sort the nodes. This single measure seems to be insufficient to determine whether two nodes are (dis)similar. The degree of a node indicates how many connections a particular node has, but it does not contain information about how these neighboring nodes are connected themselves. Therefore, additional local graph parameters are needed to better define both close and deep contacts of a given node, which are expected to estimate the ADP for the respective atom.

An extension of the node degree or the number of connections per node was introduced by Pržulj (2007 ▸). In this work, small (2–4 nodes) subgraphs, called graphlets, were introduced. Similar to counting the number of edges per node, one can also count the number of graphlets per node. Thus, by counting the graphlets (or small motifs) per node in the graph, we can extract the local topology of the node. This topological description of the nodes contains information about the number of connections as well as information about how neighboring nodes are connected.

In the work presented here, the graphlet degree vector (GDV) was used to build a multiple linear regression model to predict the distribution of protein isotropic ADPs (*B* values). It was shown that the multiple linear regression model using the GDV to predict the distribution of *B* values performs better than the linear model based on only the atomic contact number (Weiss, 2007 ▸). The multiple linear model is independent of resolution and is only based on the geometry of the model. It can be useful to predict the distribution of *B* values for macromolecular models obtained by macromolecular crystallography, cryo-EM or structure prediction (Jumper *et al.*, 2021 ▸; Baek *et al.*, 2021 ▸).

## 2. Methods

### 2.1. Graphlet degree vector

**Figure 1:**
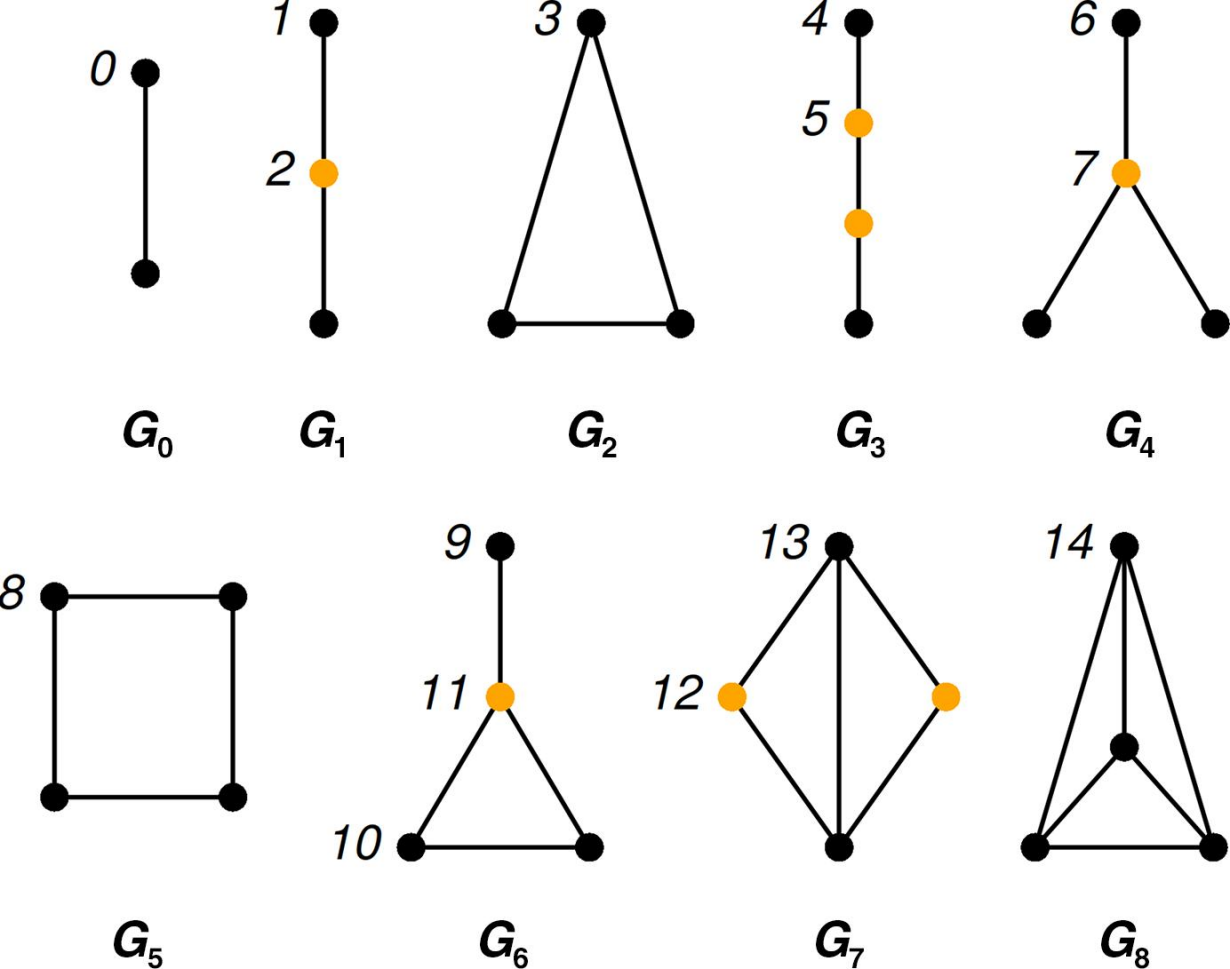
Graphlets of size 2 (*G*_0_), 3 (*G*_1_ and *G*_2_) and 4 (*G*_3_–*G*_8_). Orbits, *i.e.* topologically different nodes, are labeled 0, 1, 2, 3, …, 14.

Graphlets are small induced subgraphs of a larger graph (Fig. 1 ▸). Graphlet *G*_0_ is the smallest graphlet and contains two topologically equal nodes labeled ‘0’ (Fig. 1 ▸). Graphlet *G*_1_ has two topologically distinct nodes labeled ‘1’ and ‘2’. When two or more nodes are topologically the same, we say that they belong to the same orbit. Thus, the nodes at the ends of graphlet *G*_1_ belong to orbit *O*_1_, while the node in the middle belongs to orbit *O*_2_. In total, graphlets of size 2, 3 and 4 contain 15 topologically distinct nodes called orbits, labeled 0, 1, 2, 3, …, 14 (Fig. 1 ▸).

**Figure 2:**
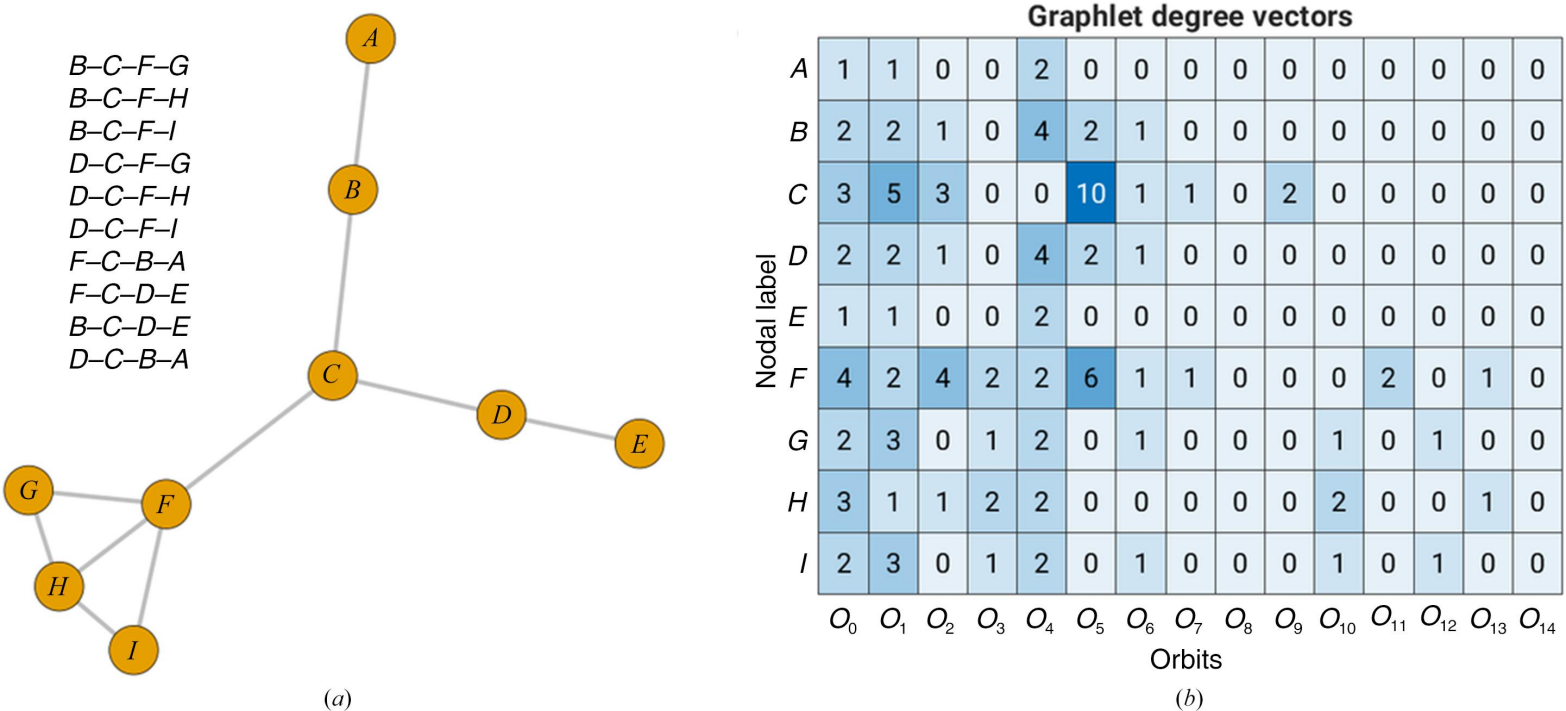
An illustration of the degree for all 15 orbits for a graph with nine nodes and ten edges. (*a*) A graph with nine nodes and ten edges; the graphlets *G*_3_ where node *C* touches orbit *O*_5_ are listed next to the graph; (*b*) the corresponding GDVs.

Visual inspection of the graph shown in Fig. 2 ▸(*a*) shows that node *C* has three edges. We obtain the same result if we count the number of edges with which node *C* touches orbit *O*_0_ (the term ‘touch’ is taken from the work of Pržulj, 2007 ▸). For example, node *C* touches orbit *O*_0_ three times, via edges *D*–*C*, *B*–*C* and *F*–*C* (Fig. 2 ▸ *a*). It follows that the degree of orbit *O*_0_ for node *C* is three. In the same way, we can now count how many times node *C* touches orbits *O*_1_, *O*_2_, …, *O*_14_. In other words, this is an extension of a node’s degree.

For illustration, the degrees for all 15 orbits for a graph with nine nodes and ten edges are shown as a colored table (Fig. 2 ▸ *b*). Thus, node *C* touches orbit *O*_1_ five times, via *E*–*D*–*C*, *A*–*B*–*C*, *G*–*F*–*C*, *H*–*F*–*C* and *I*–*F*–*C*. Node *F* touches orbit *O*_0_ four times (*C*–*F*, *G*–*F*, *H*–*F* and *I*–*F*), while it touches orbit *O*_1_ only twice (*D*–*C*–*F* and *B*–*C*–*F*). Only nodes *F*, *G*, *H* and *I* touch orbit* O*_3_: ‘triangle’. The highest degree (10) corresponds to node *C *and orbit *O*_5_. All corresponding graphlets *G*_3_, where node *C* touches orbit *O*_5_, are listed next to the graph in Fig. 2 ▸(*a*).

Therefore, for each query protein we obtain a matrix of size *N* × *M*, where *N* is the number of atoms and *M* is 15 (the total number of orbits). Thus, each element of the matrix contains the degree of a particular orbit for each node (atom).

### 2.2. Multiple linear regression

A multiple linear regression model has been used to predict the *B* values of protein atoms from the atomic GDV considering atoms as nodes of a graph, as described above. To enable comparison between the *B* values of different protein structures, the *B* values of each protein structure were independently normalized so that the mean *B* value was set to 0 and the standard deviation of the *B* value was set to 1. Multiple linear regression searches for a linear relationship between explanatory variables and the dependent variable. In this study, the explanatory variables were the components of the GDV and the dependent variable was the *B* value. Since the degree of orbits per atom can vary and it is more likely to find a higher degree for orbit *O*_0_ than for orbit *O*_14_, the columns of the matrix *N* × *M*, where *N* is the total number of atoms in the given protein and *M* is the length of the GDV, were normalized so that the mean of all columns equals 0 and the standard deviation equals 1. The multiple regression model (GDV model) with 15 explanatory variables and *N* protein atoms is written as 

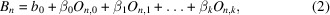

where *B*_*n*_ is the dependent variable, *n* = 1, 2, …, *N*, *b*_0_ is the intercept, *O*_*n*,*k*_, *k* = 0, 1, 2, …, 14 are explanatory variables and β_*k*_ are the coefficients of the vector of regression. The orbit *O*_0_ contains information about the number of contacts per atom. For comparison with the GDV model, a linear model (contact model) with an independent variable *O*_0_ was also used, 

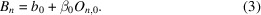

To calculate the efficiency of the contact and GDV models, the correlation between the predicted *B* values and those deposited in the PDB-REDO database (Joosten *et al.*, 2009 ▸, 2014 ▸) was calculated.

### 2.3. Software

The *R* package (version 4.2.1; R Core Team, 2022 ▸) was used for data analysis with the following packages: *orca* (version 1.1-1; Hočevar & Demšar, 2014 ▸, 2016 ▸), *netdist* (version 0.4.9100; Ali *et al.*, 2014 ▸), *bio*3*d* (version 2.4-2; Grant *et al.*, 2006 ▸), *igraph* (version 1.2.6; Csardi & Nepusz, 2006 ▸), *caret* (version 6.0-90; Kuhn, 2008 ▸), *MASS* (version 7.3-58.1; Venables & Ripley, 2002 ▸) and *invgamma* (version 1.1).

A simplified algorithm for constructing the graph and counting the orbits is presented below.

Step 1. The *bio*3*d* package is used to read the PDB file and extract the atomic coordinates.

Step 2. The distance matrix between all pairs of atoms is calculated.

Step 3. The adjacency matrix is created (a link exists if the distance is less than a certain threshold).

Step 4. The adjacency matrix as input data and the *igraph* package are used to create a graph.

Step 5. The graph from step 4 and the *orca* (inside *netdist)* package are used to count the number of orbits (degree) for each node.

The final result is a matrix of dimensions *N* × *M*, where *N* is the number of atoms and *M* is 15, with orbits *O*_0_, *O*_1_, …, *O*_14_. The *R* scripts for reading in the protein coordinates, creating the graph, counting the orbits and predicting *B* values can be found at https://github.com/jure-praznikar/Graphlets-B-value.

To order the variables in the multiple linear regression according to their importance, the *varImp* function (*R* package *caret*) was used. In general, the most important variable is the one that explains most of the variance of the response variable. The *R* function *varImp* uses the absolute value of the *t*-statistic to measure the importance of the variables.

All figures containing a ribbon representation of the 3D protein model were created using *Visual Molecular Dynamics* (Humphrey *et al.*, 1996 ▸).

## 3. Results and discussion

### 3.1. Data set

The *PISCES* protein-sequence culling server (Wang & Dunbrack, 2003 ▸) was used to obtain a Protein Data Bank identification (PDBid) list of protein structures with the following characteristics: maximum mutual sequence identity of 40%, X-ray resolution range of 1.6–2.6 Å, crystallographic *R* value less than or equal to 0.25 and protein size between 50 and 500 residues. After retrieving the PDBid list, the following filters were applied: exclusion of assemblies with more than 10 000 atoms, exclusion of proteins with missing *B* values, exclusion of assemblies with *B* values greater than 200 Å^2^, exclusion of assemblies with an extremely low *B*-value standard deviation (below 0.1) and exclusion of assemblies with low Ramachandran and rotamer *Z*-scores (less than −2). Assembly here refers to all chains identified in biological assembly 1. The Ramachandran and rotamer *Z*-score data were obtained from the PDB-REDO database available at https://pdb-redo.eu/download. The PDBid list was then used to retrieve 2107 entries from the PDB-REDO database (Joosten *et al.*, 2009 ▸, 2014 ▸).

The distribution of *B* values for each entry was analyzed using SIGD as proposed by Masmiliyeva and Murshudov (Masmaliyeva & Murshudov, 2019 ▸; Masmaliyeva *et al.*, 2020 ▸). For each entry, the SIGD parameters, namely the shape (α) and scale (β), were calculated and plotted against resolution (Supplementary Fig. S1). PDB-REDO database entries with low or high α and β values that fell outside the 95% prediction interval were excluded from further analysis. Thus, the final data set included 1957 PDB-REDO models. Since the *B* values in crystallographic models depend on the packing of atoms, symmetry-related residues were added to the PDB-REDO model. For this purpose, *WHAT IF* was used (Vriend, 1990 ▸; Rodriguez *et al.*, 1998 ▸). It adds all symmetry-related residues that possess at least one atom which makes a contact with an atom in the original protein structure. Two symmetry-related atoms are considered to be in contact when the distance between their van der Waals surfaces is smaller than 5.0 Å. Supplementary Fig. S2 shows an example of a PDB-REDO structure with symmetry-related residues used in this work.

### 3.2. Optimization of the cutoff distance

To calculate the GDV, the 3D protein model must first be converted into a graph where nodes represent protein atoms. Two nodes are connected by an edge if the respective atoms are at a distance shorter than a prescribed value, referred to below as the cutoff distance. The graph edges do not distinguish between covalently and noncovalently bonded atoms.

During crystallographic refinement, various restraints are used principally to ensure that chemically bonded atoms have similar *B* values. Similar restraints were applied to GDVs. To do so, a new smoothed value was assigned to a given atom as the sum of the current value and the average value of all neighboring nodes within a 2.0 Å radius. We need to distinguish between the cutoff distance used to create the graph and the 2.0 Å distance used in the smoothing procedure. This smoothing distance, being slightly above the length of covalent bonds, was kept constant while the optimal cutoff distance used to generate the graph was searched for.

**Figure 3:**
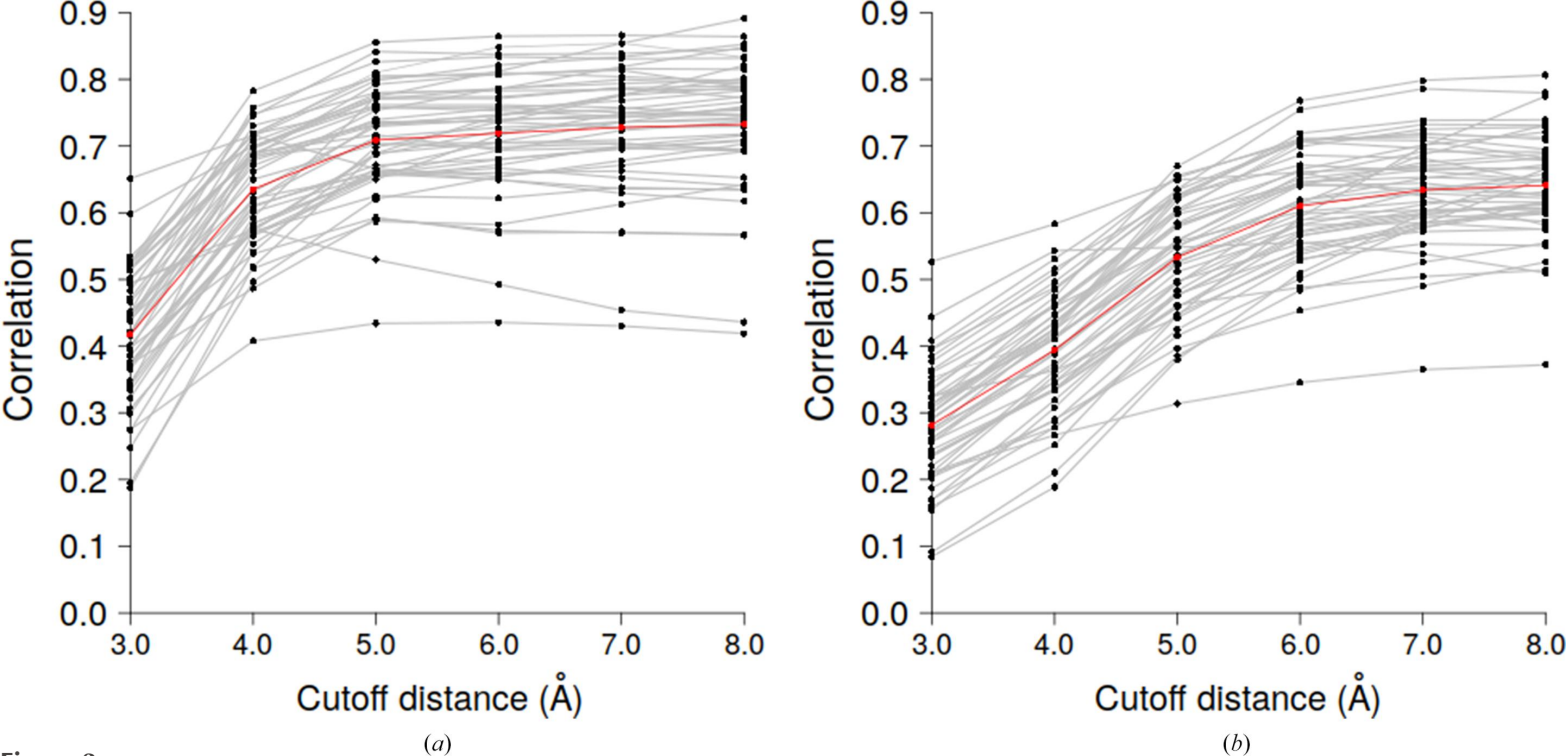
Correlation between PDB-REDO and predicted *B* values as a function of the cutoff distance for 50 randomly selected structures from our database. (*a*) GDV model, (*b*) contact model; the red line represents the average value.

To define this distance, we first randomly selected 50 entries that were used to train and validate the (multi)linear model. The model was built on 90% of the structures, which were then used to predict the test set (10% of protein structures). In the frame of tenfold cross-validation, this procedure was repeated ten times. For each entry, the correlation coefficient between the predicted and the PDB-REDO *B* values was calculated and used to find the optimal cutoff distance. Fig. 3 ▸ shows the correlation between the predicted and PDB-REDO *B* values. Its behavior was similar for all tested models, allowing us to make some conclusions.

In the case of the GDV model, the lowest correlation was found at the shortest cutoff distance that we tried, 3.0 Å (Fig. 3 ▸ *a*), while the highest correlation values were found in the interval 5.0–8.0 Å, with no significant difference in this interval. Therefore, a cutoff distance of 5.0 Å, which minimizes the calculations, was considered to be the best choice and was used in further analysis.

For comparison, we repeated the same procedure with the contact model. Here, the optimal cutoff distance (Fig. 3 ▸ *b*) was different from that for the GDV model, agreeing with the value of 7.0 Å determined previously by Weiss (2007 ▸). The overall correlation obtained by the GDV model is higher than that obtained by the contact model.

The reason why the correlation in the case of the GDV model reaches a plateau at a shorter cutoff distance is that the GDV incorporates information about ‘deep contacts’, *i.e.* a neighbor of the neighbor. For example, graphlet *G*_3_ could represent a C^α^–C^α^ wiring between two adjacent residues, the distance of which is typically ∼3.8 Å for a *trans* peptide. A quick estimate of the average distance of deep contacts lying outside the spherical radii is half of 3.8 Å, *i.e.*1.9 Å. If we add the GDV model cutoff distance of 5.0 Å and the estimated deep contact distance of 1.9 Å, we obtain a distance of 6.9 Å, which is consistent with the cutoff distance of the contact model.

### 3.3. Prediction of the *B*-value distribution

**Figure 4:**
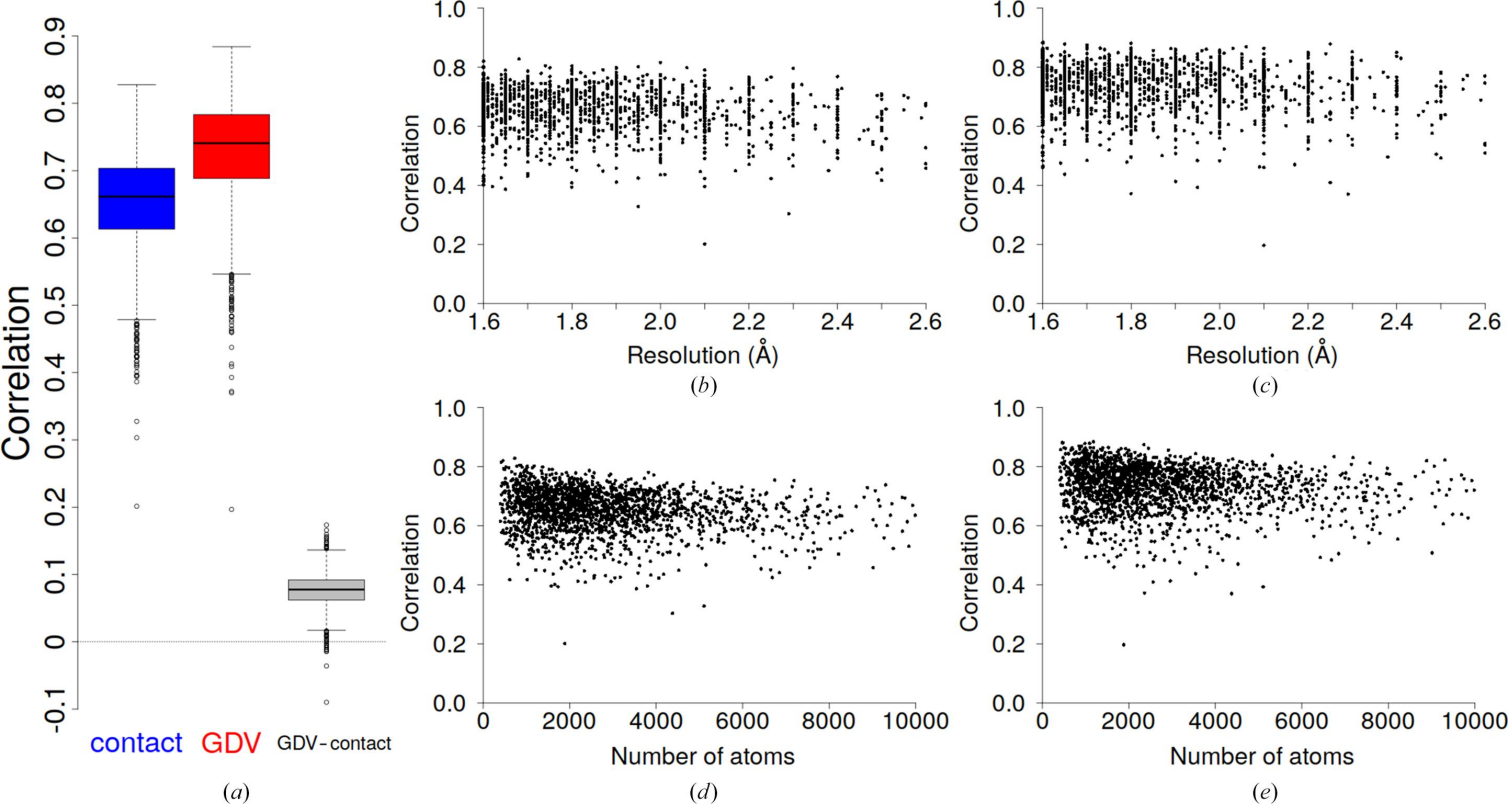
(*a*) Box plots of correlations between PDB-REDO and predicted *B* values for the contact and GDV models. The pairwise delta correlation GDV–contact box plot represents the difference between the GDV and contact models. (*b*) Correlation versus resolution for the contact model, (*c*) correlation versus resolution for the GDV model, (*d*) correlation versus number of atoms (protein size) for the contact model and (*e*) correlation versus number of atoms (protein size) for the GDV model.

Fig. 4 ▸(*a*) shows a box plot of all correlation values between PDB-REDO and predicted *B* values for 1957 entries using the contact model defined by equation (3) and the GDV model defined by equation (2), each with its own cutoff distance: 7.0 and 5.0 Å, respectively. Both models, contact and GDV, were validated using the same procedure (tenfold cross-validation) as described in §3.2.

The GDV model performed better than the contact model, with the average correlation increased by 0.08 (0.73 versus 0.65) and the largest correlation increased by 0.17. The average value does not indicate in how many cases the GDV model was better compared with the contact model. Therefore, the delta correlation (GDV–contact) was calculated, defined as the GDV–model correlation minus the contact–model correlation. The box plot shows (Fig. 4 ▸ *a*) that the GDV model performs better than the contact model for the vast majority, 1943 (or 99.3%), of the PDB-REDO entries used in the tests.

Thus, the introduction of additional variables and information in comparison with the contact model improves the results. It should be emphasized that both models predict the distribution of *B* values, *i.e.* normalized values, and not the absolute *B* values (in Å^2^). Their rescaling to predict non-normalized *B* values is also possible, but only to some extent. To do so, the mean *B* value and the standard deviation of the *B* value of the model are needed. These values are resolution-dependent (Carugo, 2018*b* ▸; Masmaliyeva & Murshudov, 2019 ▸; see also Supplementary Fig. S3), and the width of the distribution for a given resolution is quite large. For example, at a resolution of 2.0 Å the mean *B* value ranges from 15 to 50 Å^2^ and the standard deviation ranges from 5 to 20 Å^2^, and using an incorrect combination of these values may result in wrongly predicted absolute *B* values.

The data set used in this study contains protein structures that have been solved at different resolutions and have quite different sizes. The correlation versus resolution plot and the correlation versus the number of atoms show that the accuracy of both the contact and GDV models does not depend on these parameters (Fig. 4 ▸). This is expected since the model is fully based on the molecular geometry. Thus, we can assume that the *B* values are not completely independent parameters but are related to the atomic coordinates: the molecular geometry. The first studies to indicate that *B* values are not completely independent parameters were presented by Halle (2002 ▸) and Weiss (2007 ▸). The former study showed that *B* values are inversely proportional to contact density, while the latter study showed that there is a linear relationship between atom contact numbers and *B* values. The GDV, or rather the graph, is also based on atomic coordinates. Thus, this study supports the assumption that the *B* values are not completely independent of the coordinates.

### 3.4. Final (multi)linear model

The final contact model built on all data, *i.e.* all atoms of all PDB-REDO entries (∼5.8 × 10^6^ atoms), is given as

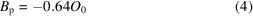

and the GDV model is given as

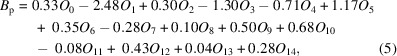

where *B*_p_ refers to the normalized predicted *B* value and *O*_0_, *O*_1_, … *O*_14_ are the degrees of node orbits. Note that the intercept value in equation (4) is 0 and the linear regression coefficient is equal to −0.64, which means that the larger the number of contacts the lower the *B* value (Halle, 2002 ▸; Weiss, 2007 ▸).

**Figure 5:**
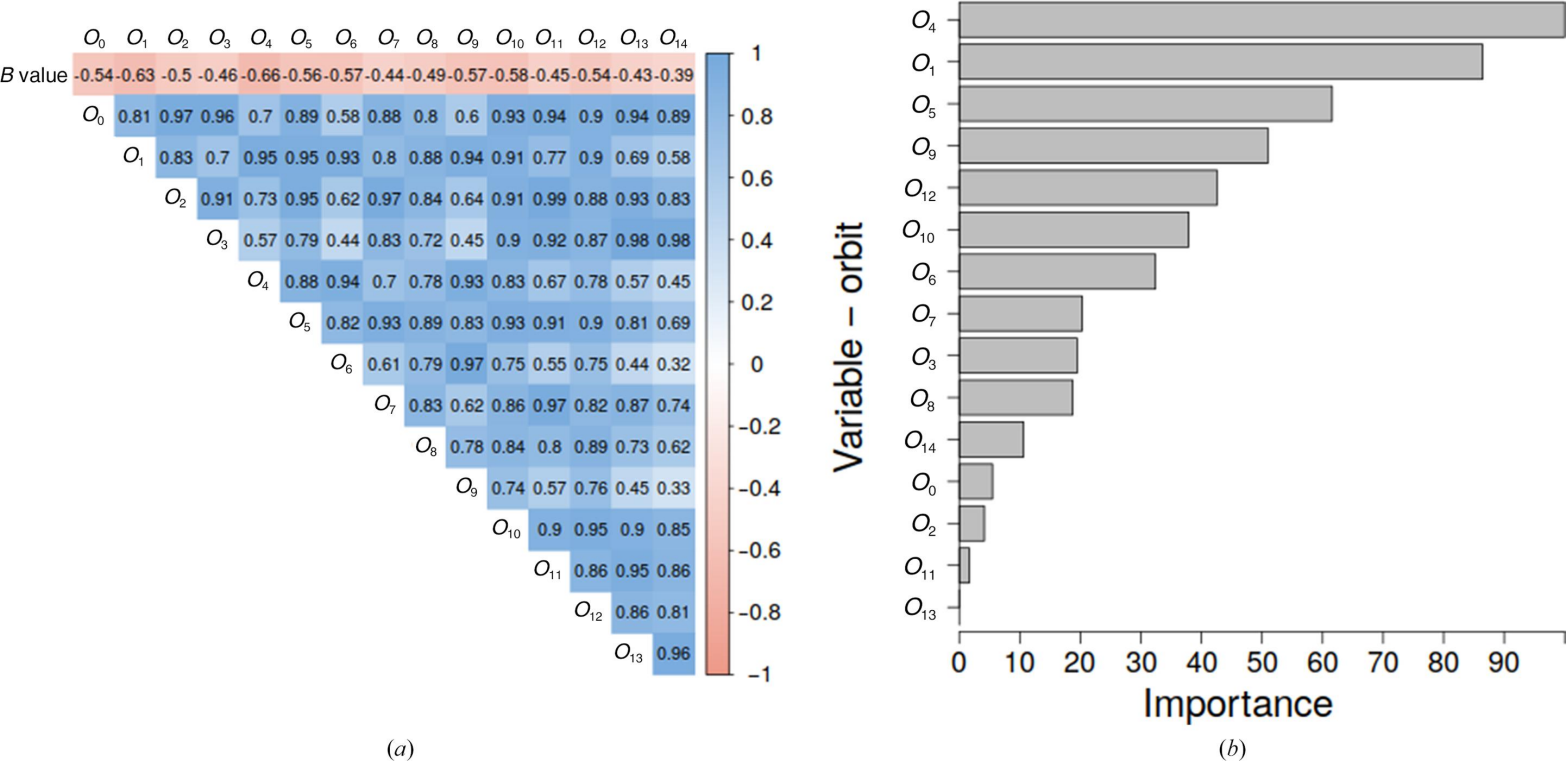
(*a*) Correlation matrix. Pairwise correlation of *B* value and orbits *O*_0_, *O*_1_, … *O*_14_. (*b*) Variable importance of the GDV model. The importance of the variables is normalized so that the most important variable has a value of 100.

A linear model (equation 4) is easier to interpret than a multiple linear model (equation 5), especially since its variables are highly correlated. Indeed, the correlation matrix (Fig. 5 ▸ *a*) shows that there is a high collinearity between orbits and that all orbits are negatively correlated with the *B* value. The *B* value is most negatively correlated with orbits *O*_1_ (−0.63) and *O*_4_ (−0.66). Therefore, instead of analyzing the magnitude and the sign of the regression coefficients in equation (5), an analysis of the most important variables was performed. The first three most important variables are *O*_4_, *O*_1_ and *O*_5_ (Fig. 5 ▸ *b*). These three orbits correspond to two graphlets, *G*_1_ and *G*_3_ (Fig. 1 ▸). It is interesting to note that orbit *O*_0_ (the number of contacts) appears to be one of the less important variables; however, it is highly correlated with several other orbits. This suggests that the types of connections of neighboring atoms are more important than the number of contacts in itself. The first three most important variables (*O*_4_, *O*_1_ and *O*_5_) correspond to the ‘unbranched’ graphlets *G*_1_ and *G*_3_, while the next four important variables are *O*_9_, *O*_12_, *O*_10_ and *O*_6_, which correspond to the ‘branched’ graphlets, namely *G*_4_, *G*_6_ and *G*_7_ (Fig. 1 ▸), and thus contain information about the internal connection between nodes.

### 3.5. The bimodal distribution of *B* values and its relationship to normalization

Approximately 15% of PDB structures exhibit multimodality of *B* values (Masmaliyeva *et al.*, 2020 ▸), and attention should be paid to how these *B* values are normalized. Two examples from our data set that have a bimodal distribution of *B* values are shown in Supplementary Fig. S4. The performance of the GDV model and its relationship to the normalization of *B* values is presented below.

#### 3.5.1. A heterotrimeric protein

**Figure 6:**
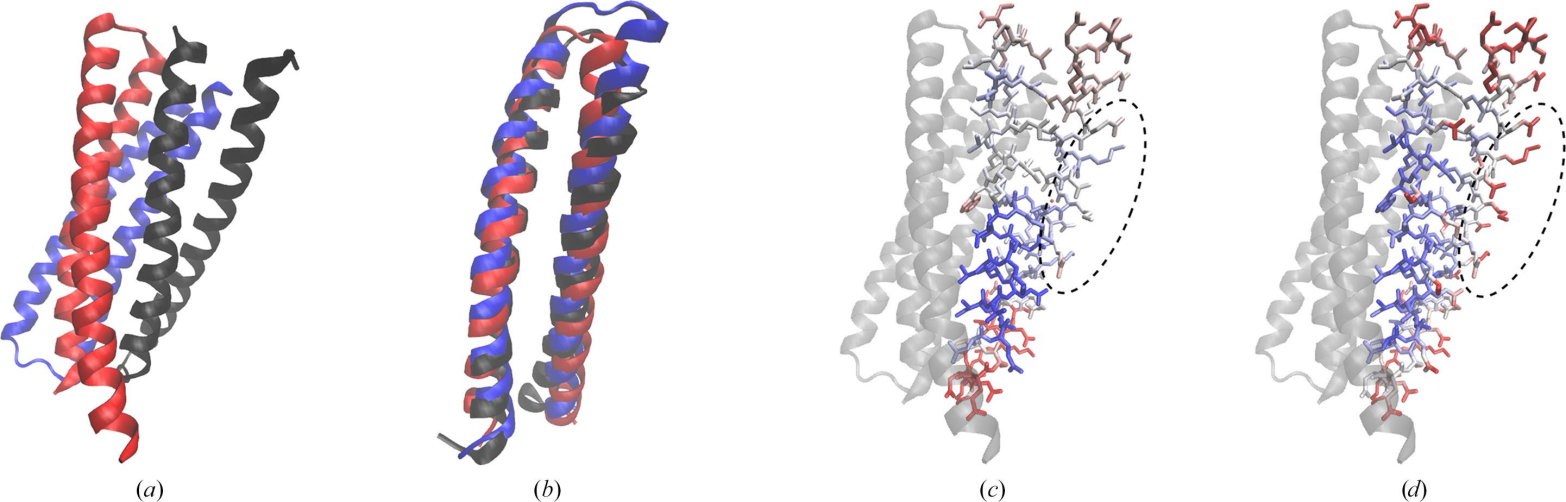
(*a*) An *ABC*-type heterotrimeric protein; chains *A*, *B* and *C* are in black, blue and red, respectively. (*b*) Aligned chains. (*c*) Chain *A* in the red–white–blue color scale according to the *B* values from the PDB-REDO model and scaled with the minimum value equal to −1 standard deviation and the maximum value equal to +1 standard deviation. Chains *B* and *C* are shown as ribbons in gray. The dashed ellipse marks solvent-exposed side chains located in the middle of the helix. (*d*) The same as (*c*) with the model colored according to the predicted *B* values.

A detailed examination of the results of the contact and GDV models revealed that the lowest correlation (∼0.20) for both models occurred in the case of PDB entry 7upo, which is also seen as an outlier in the box plot (Fig. 4 ▸ *a*). The structure of this obligate *ABC*-type heterotrimeric protein is a *de novo* design determined at 2.1 Å resolution (Bermeo *et al.*, 2022 ▸). Each monomer consists of two helices of about 35 residues in length connected by short loops, with two loops on the same side (chains *A* and *B*) and one loop on the opposite side (chain *C*) of the heterotrimer (Fig. 6 ▸ *a*). Visual inspection shows that chains *A*, *B* and *C* have a similar spatial structure (Fig. 6 ▸ *b*). The template-modeling score (TM-score; Zhang & Skolnick, 2005 ▸) of the aligned chains ranges from 0.64 to 0.79, while the pairwise sequence identity between chains *A*, *B* and *C* is less than 35% (Supplementary Table S1).

**Figure 7:**
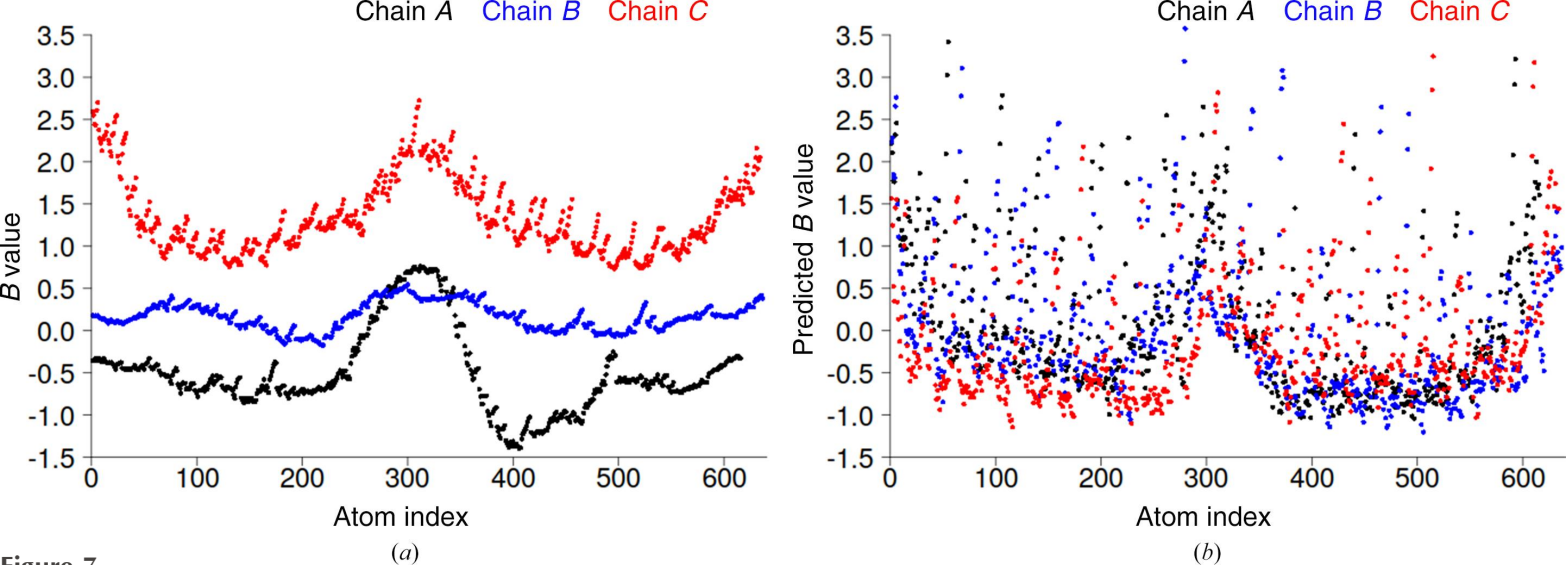
*B* values of an *ABC*-type heterotrimeric protein (PDB entry 7upo). (*a*) PDB-REDO and (*b*) predicted *B* values. *B* values were normalized so that the mean *B* value was set to 0 and the standard deviation of the *B* value was set to 1.

Analysis of the *B* values shows that the chains with the same orientation (chains *A* and *B*) have similar *B* values, while chain *C*, which has the opposite orientation, has values that are significantly higher (Fig. 7 ▸ *a*). The *B* values of chain *A* vary rather smoothly over neighboring atoms compared with those of chain *B* and especially those of chain *C*. The correlation coefficients between the predicted and PDB-REDO *B* values are 0.48, 0.46 and 0.52 for chains *A*, *B* and *C*, respectively (Supplementary Fig. S5). Thus, the accuracy of the predicted *B* values per each chain was modest (∼0.50), and was low (∼0.20) for all three chains considered together.

Another notable difference between the predicted and deposited *B* values is that the covalently bonded atoms of the PDB-REDO structure have very similar *B* values (Fig. 7 ▸ *a*), whereas the predicted *B* values vary considerably (Fig. 7 ▸ *b*), *i.e.* they are less smoothed. In general, for all three chains we can see that the PDB-REDO and predicted *B* values are higher at the chain termini and in the loop region. Individual high predicted *B* values correspond to side-chain atoms exposed to solvent (Fig. 6 ▸ *d*), as expected, while solvent-exposed side chains located in the middle of the helix of the PDB-REDO structure do not have high *B* values (Fig. 6 ▸ *c*). A brief examination of the crystal contacts revealed that the solvent-exposed side chains in the middle of the helix, indicated by a dashed ellipse in Figs. 6 ▸(*c*) and 6 ▸(*d*), are not involved in a large number of crystal contacts.

The main reason for the significant difference between global (asymmetric unit) and local (chain) accuracy is the magnitude of the *B* values. When the chains or domains have significantly different mean *B* values, it is more reasonable to perform normalization for each unit (chain or domain) separately and then calculate the correlation between the predicted and deposited *B* values to evaluate the efficiency of the GDV model. It is interesting to note that chains *A*, *B* and *C* of the examined heterotrimeric protein correspond to three translation–libration–screw (TLS) groups. Thus, as an alternative to manual selection, it is also possible to perform normalization according to the predefined (large) TLS groups (Schomaker & Trueblood, 1968 ▸).

#### 3.5.2. Two monomers in the asymmetric unit

**Figure 8:**
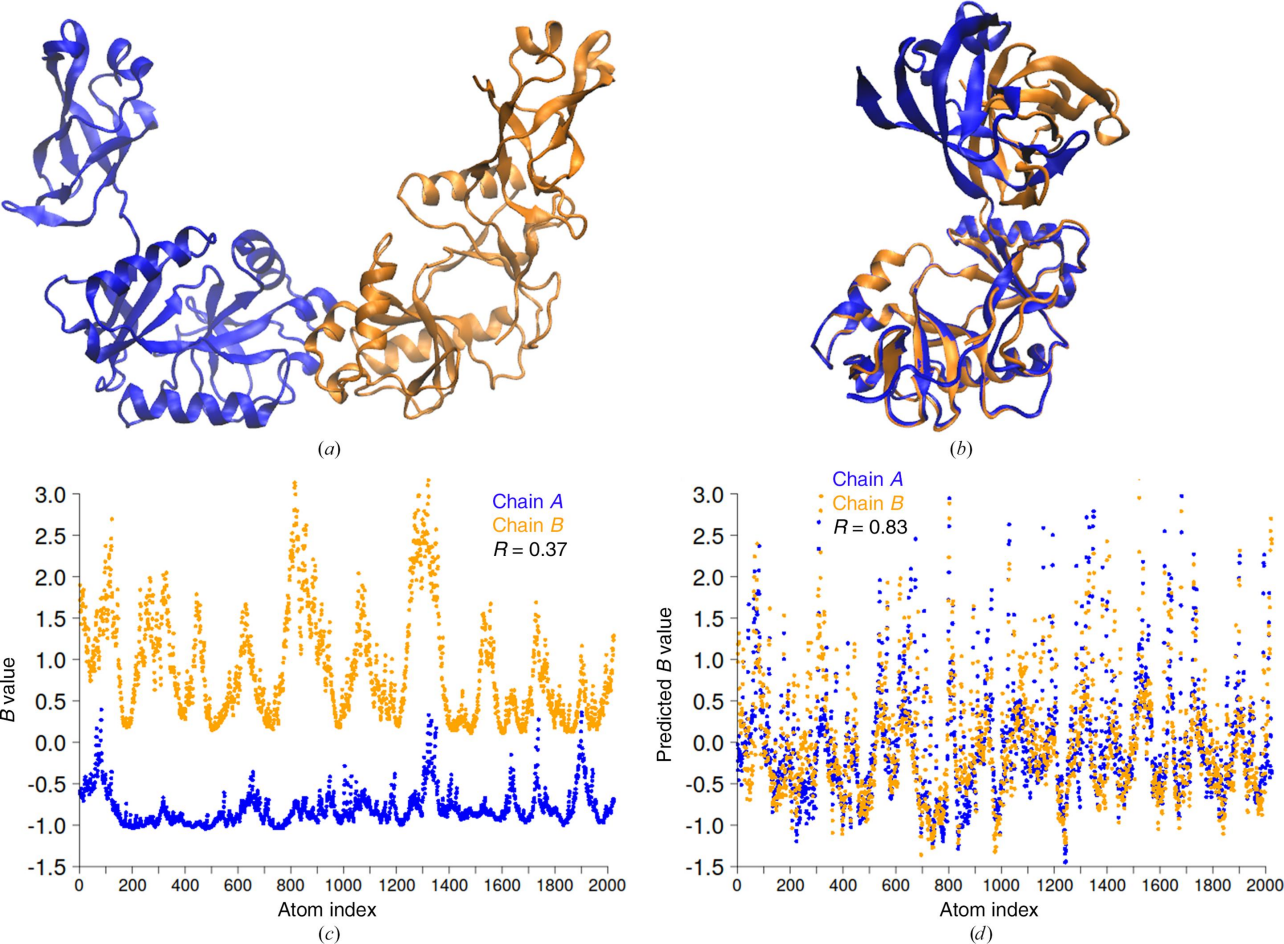
(*a*) Two monomers of the enzyme–adenylate complex in the asymmetric unit (PDB entry 4d05); chain *A* is in blue and chain *B* is in orange. (*b*) Superimposed monomers of the enzyme–adenylate complex. (*c*) PDB-REDO *B* values. (*d*) Predicted *B* values. *B* values were normalized so that the mean *B* value was set to 0 and the standard deviation of the *B* value was set to 1.

The structure of the obligate enzyme–adenylate complex (PDB entry 4d05), determined at 1.65 Å resolution (Williamson *et al.*, 2014 ▸), was obtained in space group *C*2 with two monomers per asymmetric unit (Fig. 8 ▸ *a*). This protein has an adenylation domain (AD domain) and an oligonucleotide-binding domain (OB domain). Superposition of the two monomers using the larger AD domain for alignment shows that they have different conformations (Fig. 8 ▸ *b*). When the domains are aligned separately, the root-mean-square deviation (on C^α^ atoms) for each domain is below 1 Å. Thus, the short contacts (∼5 Å) remain very similar when comparing the two monomers. This suggests that the predicted *B* values should also be similar. Indeed, the correlation of the predicted *B* values between the monomers is 0.83 (Fig. 8 ▸ *d*). On the other hand, the correlation of the deposited *B* values between the monomers is only 0.37 (Fig. 8 ▸ *c*). Also, the magnitude of the *B* values in chain *B* is much higher compared with that in chain *A*. The correlation between the PDB-REDO and predicted *B* values was 0.65 for chain *A*, while the correlation for chain *B* was significantly lower at ∼0.40 (Supplementary Fig. S6). It should be noted that the authors (Williamson *et al.*, 2014 ▸) used chain *A*, defined as biological assembly 1, as a reference for further discussion because it has more complete density and a lower mean *B* value compared with chain *B* (biological assembly 2).

This and the previous example of a heterotrimeric protein demonstrate that if the *B* values in the protein model have a multimodal distribution and we want to evaluate the performance of the GDV model, the *B* values should be normalized with respect to the modes. The modes or clusters of *B* values in a given protein model are of course case-dependent and correspond, for example, to chains, domains or large TLS groups.

### 3.6. Application to electron microscopy structures

Finally, the contact and GDV models (equations 4 and 5) were tested on several structures determined by cryo-EM at a resolution higher than 2.5 Å and containing fewer than 10 000 non-H atoms per independent component. Previously, Wlodawer *et al.* (2017 ▸) pointed out that the *B* values in almost all deposited cryo-EM models were meaningless. All cryo-EM structures used in our study were deposited in the period from 2019 to 2022, and it appears that quality control has improved.

**Figure 9:**
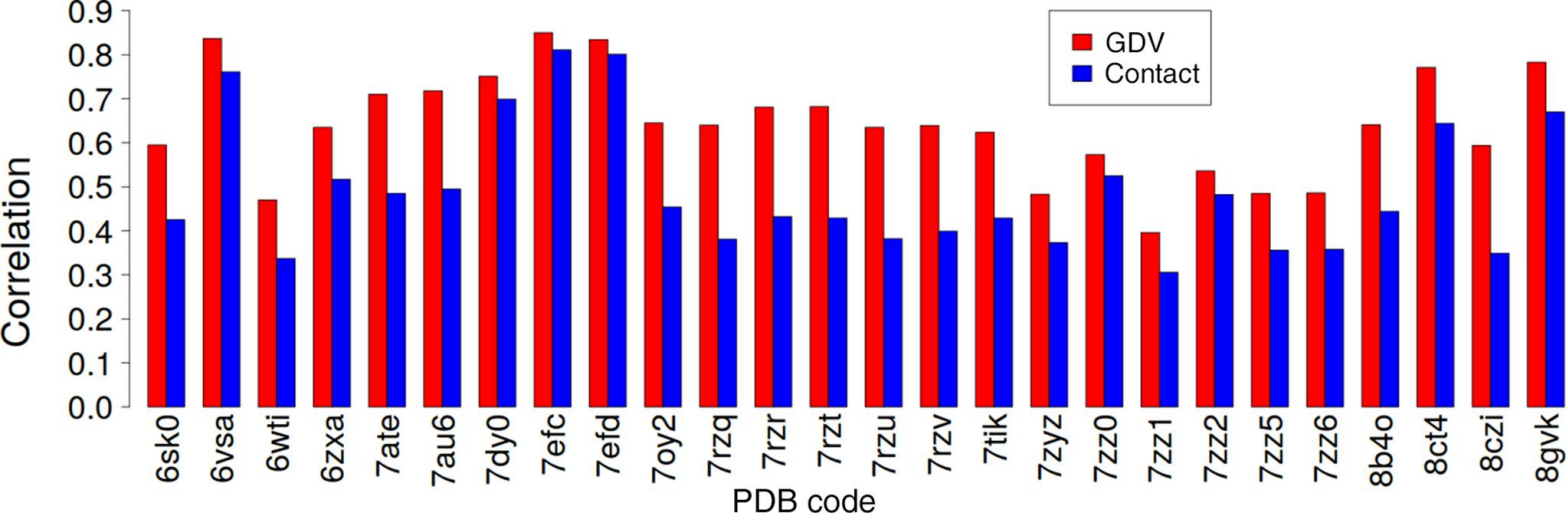
The correlation coefficient between the predicted and deposited *B* values for 26 cryo-EM structures. Correlations for the GDV model and the contact model are shown in red and blue, respectively.

The correlation coefficient between the predicted and the deposited *B* values for the contact and GDV models is shown in Fig. 9 ▸. In the case of the GDV model, the average correlation between the PDB and predicted *B* values was ∼0.64 for 26 cryo-EM structures, which is better on average by ∼0.15 than for the contact model. The largest difference was for PDB entry 7rzq, with correlations of 0.64 and 0.38, respectively. One can speculate that for this apparently difficult structure the GDV method is still capable of predicting some information about atomic mobility, even if far from being perfect, while the contact model essentially fails.

## 4. Conclusions

This study presents an improved approach for predicting the distribution of *B* values over a protein structure. This approach uses the graphlet degree vector (GDV). The components of the GDV describe the complexity of the wiring for a given atom in a macromolecule considering not only its number of direct contacts but also information about the contacts of its neighbors. A multiple linear regression model was developed using GDV components as explanatory variables. The tests showed that this model outperforms the linear model (Weiss, 2007 ▸) based only on direct atomic contacts. Since the GDV model is built on purely geometric considerations, the *B* values are not completely independent parameters, and its performance does not depend on the resolution of the experimental data.

A disagreement between the predicted and the experimentally obtained *B* values may be attributed both to imperfections in the method and the presence of static errors in the experimental values, since the deposited models also reflect variation of the structures over samples. In particular, this second component is responsible for the dependence of the *B* value on the resolution of the data. An obvious imperfection of the suggested model is the difficulty in obtaining the *B* values on an absolute scale and not on the normalized scale.

Despite the incompleteness and imperfection of such modeling, it is the dynamic aspect of protein structure that most interests structural biologists, and this improved method to predict it can help to both obtain an idea about atomic mobility and provide one with starting values for accurate *B*-value refinement. It should be mentioned that a certain degree of caution is required when using deposited or predicted crystallographic *B* values to analyze the dynamics of the protein structure, as the *B* values of the exterior residues may be biased by close crystal contacts. Nevertheless, the GDV model is an important complementary tool to structure-prediction software such as *AlphaFold* and *RoseTTAFold* (Jumper *et al.*, 2021 ▸; Baek *et al.*, 2021 ▸).

Future research could focus on using the model to validate protein models deposited in the PDB and also on including non­protein atoms; for example, nucleic acids and ligands. The low correlation between predicted and deposited *B* values could be due to either a multimodal distribution of *B* values or a partially incorrect model. The former means that normalization per domain/chain or TLS group should be reconsidered. The latter means that the positions of some atoms or loops should be corrected. Therefore, future work will consider applying the GDV model to the entire PDB and identifying potentially incorrectly modeled regions in protein models. However, local errors in protein structure are not the only source of differences between deposited and predicted *B* values. The cause of the discrepancy between deposited and predicted *B* values can also be radiation damage, for example (Gerstel *et al.*, 2015 ▸; Shelley *et al.*, 2018 ▸).

In addition to validating and applying the model to large databases, the model can be improved by using advanced prediction methods or by combining the GDV model with other proven approaches. For example, the hierarchical disorder model introduced by Pearce & Gros (2021 ▸), which uses a set of TLS parameters to represent structural disorder at different structural levels, can be combined with the GDV model to create a multivariate multiple linear model in which the response variables are partial *B* values at the chain, secondary-structure, residue and atom levels. An alternative way to further analyze the *B* values is to also cluster *B* values in search of typical vectors for main chains, side chains, inner or outer atoms.

## Supplementary Material

Supplementary Figures and Table. DOI: 10.1107/S2059798323009142/di5068sup1.pdf
