# Supplementary material for: Using graphlet degree vectors to predict atomic displacement parameters in protein structures
Source: Acta Crystallogr D Struct Biol. 2023 Nov 21;79(Pt 12):1109–19. doi: 10.1107/S2059798323009142 (PMC10833351; doi:10.1107/S2059798323009142)
Supplement: Supplementary file 1 [file d-79-01109-sup1.pdf]

Acta Cryst D STRUCTURAL BIOLOGY

Volume 79 (2023)

Supporting information for article:

Using graphlet degree vectors to predict atomic displacement parameters in protein structures

Jure Pražnikar

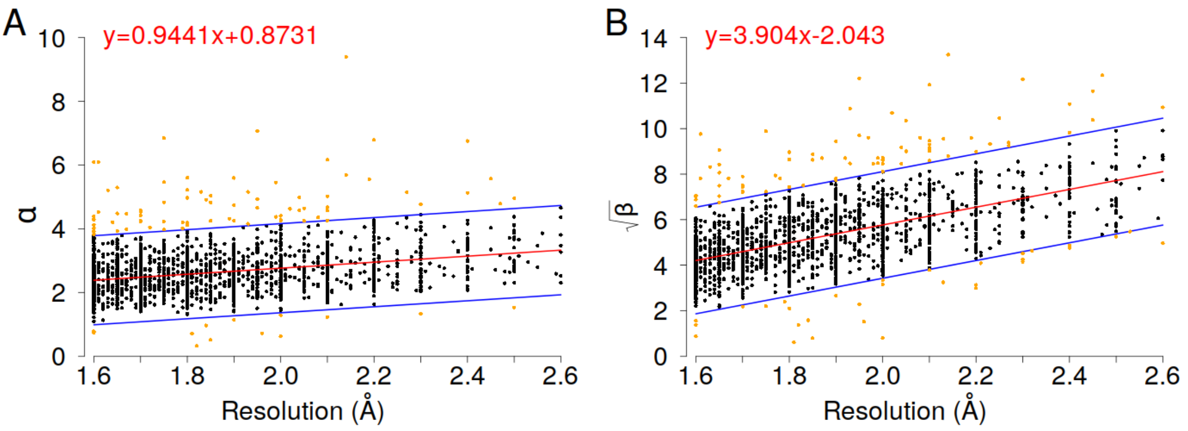

Figure S1 Dependence of the parameters of the SIGD on resolution. The linear fit (red line) of the shape (α) and scale ($\beta^{0.5}$) parameters against resolution is shown. The blue lines correspond to the upper and lower prediction intervals at a confidence level of 95%. The data points falling outside the 95% prediction interval are in orange; they were excluded from the further analysis.

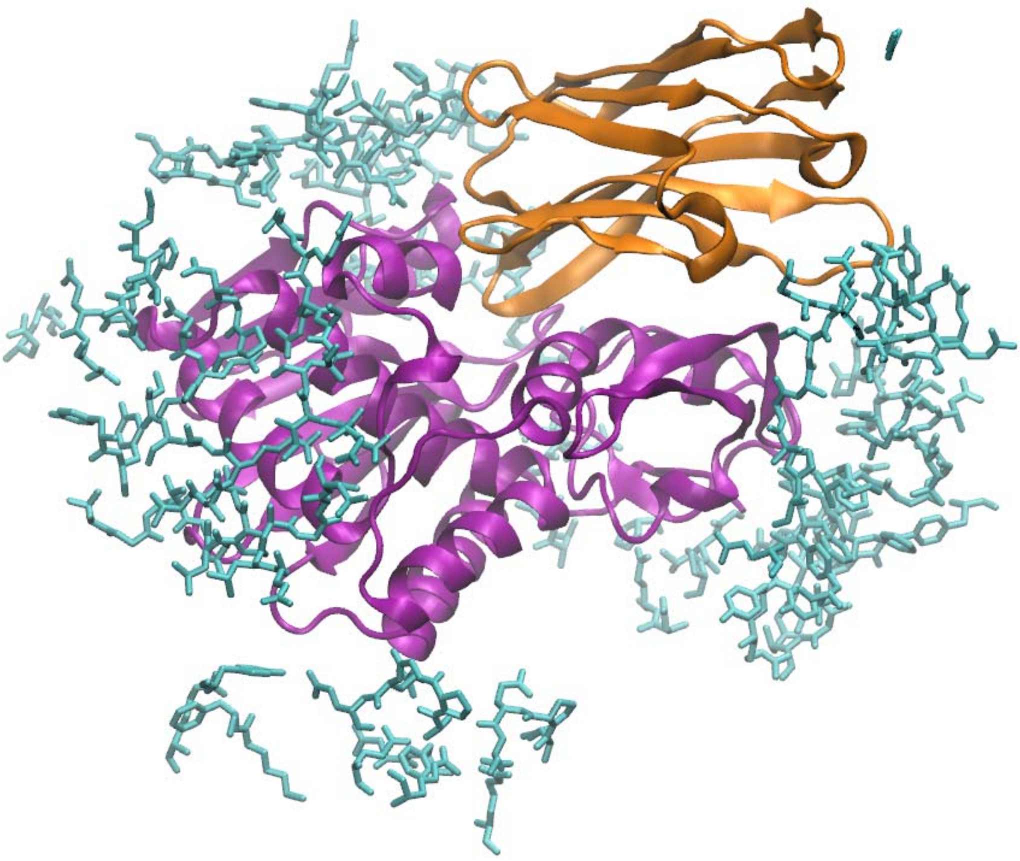

Figure S2 Model with symmetry-related residues. PDB-REDO entry (PDBid: 6obe) is shown as a ribbon, chain A in purple and chain B in orange, while symmetry-related residues are shown as cyan sticks.

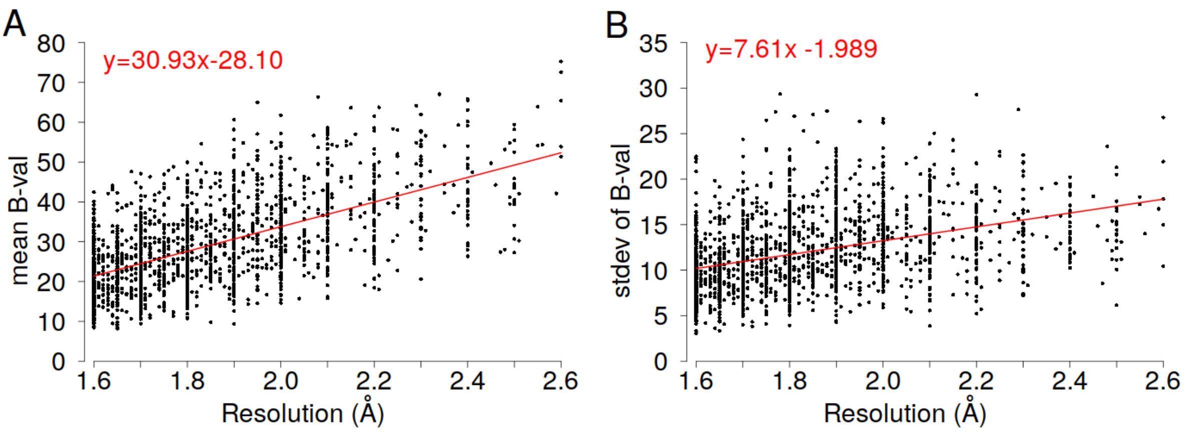

Dependence of the mean and standard deviation of the B-value on resolution. The linear regression line of best fit is represented by the red line.

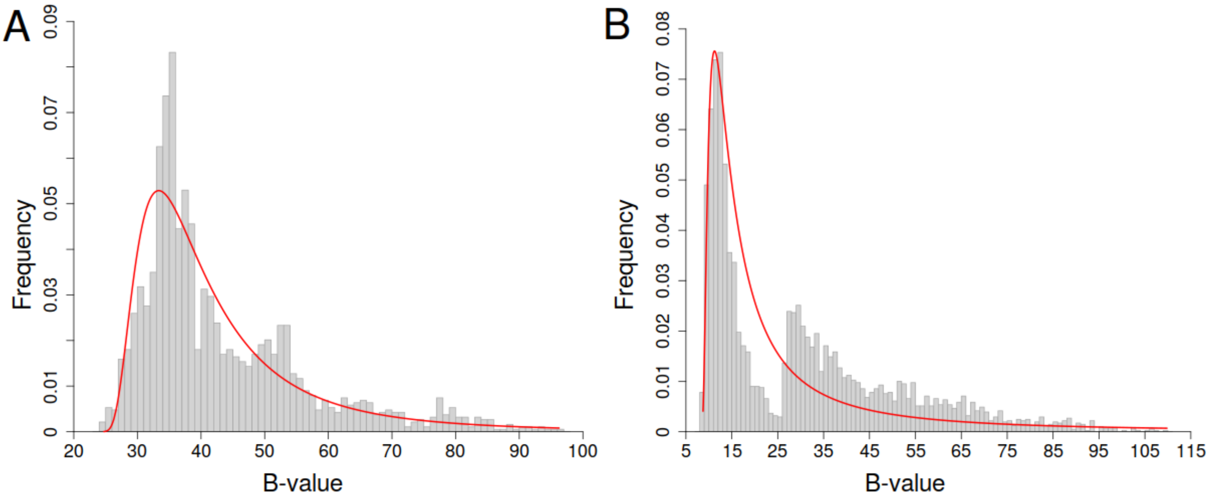

Figure S3 B-value distribution and fitted SIGD (red color) are shown; (A) PDBid: 7upo and (B) PDBid: 4d05.

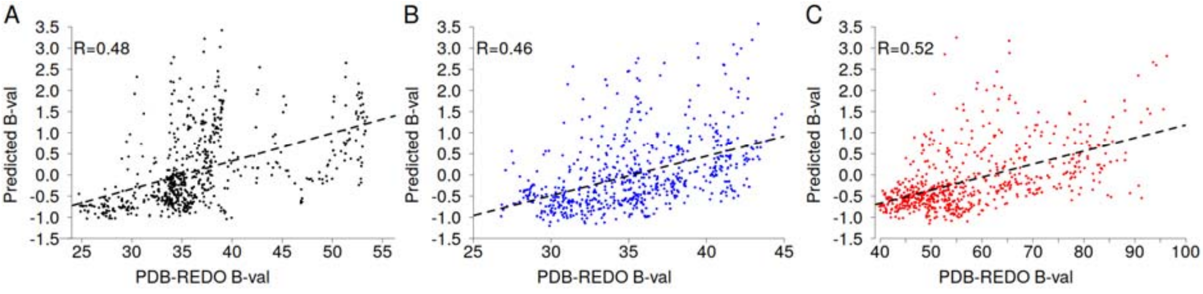

Figure S4 Scatter plot of the predicted versus PDB-REDO B-values of ABC-type heterotrimeric protein (PDBid: 7upo); (A) chain A, (B) chain B and (C) chain C.

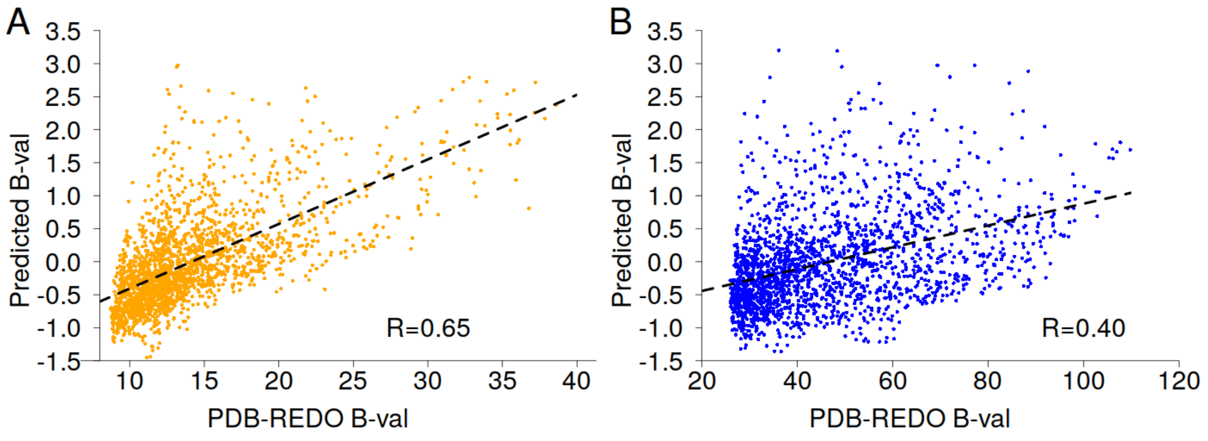

Figure S5 Scatter plot of the predicted versus PDB-REDO B-values of the enzyme-adenylate complex (PDBid: 4d05); (A) chain A and (B) chain B.

Table S1 Pairwise comparison of the heterotrimeric chains: A, B, and C (PDBid: 7upo).

The upper right triangle is the pairwise template modeling score (or TM-score), the bottom left is the pairwise sequence identity (%).

chain	A	B	C
A	/	0.79	0.71
B	30	/	0.64
C	32	18	/
